# Small sequence variations between two mammalian paralogs of the small GTPase SAR1 underlie functional differences in coat protein complex II assembly

**DOI:** 10.1074/jbc.RA120.012964

**Published:** 2020-05-01

**Authors:** David B. Melville, Sean Studer, Randy Schekman

**Affiliations:** Department of Molecular and Cell Biology, Howard Hughes Medical Institute, University of California, Berkeley, Berkeley, California, USA

**Keywords:** COPII, GTPase, apolipoprotein, intracellular trafficking, membrane trafficking, secretion, SAR1A, SAR1B, SEC23, SEC31

## Abstract

Vesicles that are coated by coat protein complex II (COPII) are the primary mediators of vesicular traffic from the endoplasmic reticulum to the Golgi apparatus. Secretion-associated Ras-related GTPase 1 (SAR1) is a small GTPase that is part of COPII and, upon GTP binding, recruits the other COPII proteins to the endoplasmic reticulum membrane. Mammals have two SAR1 paralogs that genetic data suggest may have distinct physiological roles, *e.g.* in lipoprotein secretion in the case of SAR1B. Here we identified two amino acid clusters that have conserved SAR1 paralog–specific sequences. We observed that one cluster is adjacent to the SAR1 GTP-binding pocket and alters the kinetics of GTP exchange. The other cluster is adjacent to the binding site for two COPII components, SEC31 homolog A COPII coat complex component (SEC31) and SEC23. We found that the latter cluster confers to SAR1B a binding preference for SEC23A that is stronger than that of SAR1A for SEC23A. Unlike SAR1B, SAR1A was prone to oligomerize on a membrane surface. SAR1B knockdown caused loss of lipoprotein secretion, overexpression of SAR1B but not of SAR1A could restore secretion, and a divergent cluster adjacent to the SEC31/SEC23-binding site was critical for this SAR1B function. These results highlight that small primary sequence differences between the two mammalian SAR1 paralogs lead to pronounced biochemical differences that significantly affect COPII assembly and identify a specific function for SAR1B in lipoprotein secretion, providing insights into the mechanisms of large cargo secretion that may be relevant for COPII-related diseases.

## Introduction

ER-to-Golgi protein trafficking is a key checkpoint in sorting of proteins for secretion. Approximately one-third of all proteins are first assembled in the ER and then sorted to other destinations by COPII. Of the five core COPII proteins, the first to arrive at the ER membrane is SAR1. SAR1 is a small GTPase with an amphipathic helix that inserts into the membrane when SAR1 is in the GTP-bound state. SAR1 then recruits the remainder of the COPII complex, SEC23/24 and SEC13/31 heterodimers, to the ER membrane (1–5). It is thought that regulation of the GTPase activity of SAR1 is important for large cargo selection (6). Thus, SAR1 has two important roles: recruitment of the other COPII proteins and controlling the timing of COPII budding with its GTPase cycle.

Given its essential nature, it is perhaps not surprising that SAR1 is extremely well conserved throughout evolution. The protein sequence of yeast and human SAR1 is either identical or strongly similar for ∼80% of the amino acid sequence, despite the fact that humans have greatly different secretory requirements from yeast. One area of divergence is that many invertebrates have only one paralog of SAR1, whereas mammals and most vertebrates have two. It is possible that these two paralogs have evolved unique functions to compensate for the diverse secretory needs of different cell types.

Genetic data provide some evidence that the two SAR1 paralogs, SAR1A and SAR1B, have divergent roles. For example, loss of SAR1B leads to chylomicron retention disease/Anderson's disease, which results in inability to transport newly synthesized chylomicrons out of intestinal epithelial cells (7–12). A similar phenotype has been observed in zebrafish with loss of SAR1B (13) but not the more SAR1A-like SAR1AB. In cell culture, SAR1B knockout in chylomicron-secreting Caco-2/15 cells disrupts lipid homeostasis and induces oxidative stress and inflammation (14, 15). SAR1A disruption produces a similar phenotype but to a lower degree than SAR1B (14). Both SAR1 paralogs are highly expressed in the intestine, and SAR1A expression increases in chylomicron retention disease/Anderson's disease patients, but this increase is insufficient to compensate for loss of SAR1B (8). Taken together, these data suggest that the SAR1 paralogs have overlapping and unique functions in cells and likely differ biochemically; however, little is known about the biochemical differences between SAR1A and SAR1B.

Here we identify two divergent clusters of conserved sequence differences between the two SAR1 paralogs. We find that a GTP-adjacent cluster alters GTP loading activity and direct interactions with SEC31A. We find that a second cluster in an apical α-helix causes SAR1B to bind more efficiently to SEC23 and SAR1A to homodimerize. We find that the apical α-helix is necessary and sufficient for more efficient rescue of lipoprotein secretion by SAR1B than SAR1A. These data present clear biochemical differences between the two paralogs that provide a possible explanation for the differences seen in genetic data.

## Results

### SAR1A and SAR1B have two clusters of divergent amino acids

**Figure 1.**
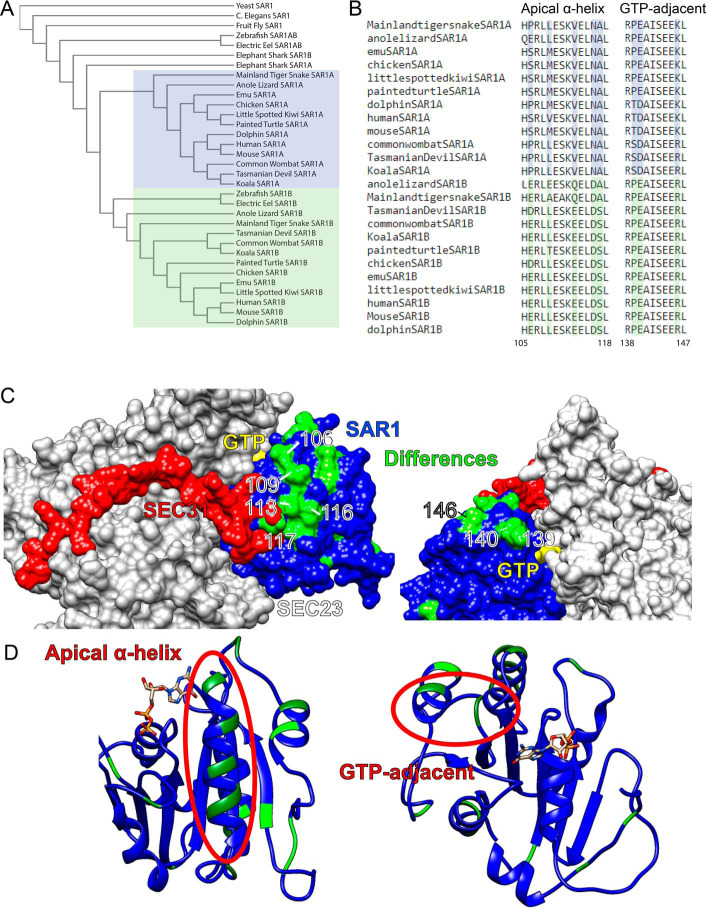
**The evolutionary conservation of SAR1 paralogs.** *A*, phylogenetic guide tree of SAR1 paralogs in three species each of invertebrates, fish, reptiles, birds, marsupials, and nonmarsupial mammals. *B*, sequence alignment of apical α-helix and GTP-adjacent clusters of divergent amino acids. *C*, structure of human SAR1A and SAR1B modeled onto yeast SEC23 (*gray*)/SAR1(*blue*)/SEC31(*red*) with divergent amino acids highlighted in *green. D*, ribbon model of SAR1 with divergent regions *highlighted*.

There are only 20 of 198 divergent amino acids that distinguish human SAR1A and SAR1B. Because highly conserved amino acid residues tend to be functionally important (17), we first wanted to compare how the SAR1A/B divergence appears in evolutionary history and determine which divergent amino acids are most conserved. We retrieved the amino acid sequences from the Ensembl database (18) and compared the sequences using Clustal Omega (19). We found that in reptiles, birds, and mammals, there were conserved distinct SAR1A and SAR1B paralogs, whereas in some fish, such as zebrafish, there was a distinct SAR1B allele and a more intermediate SAR1AB allele closer to the invertebrate ancestral gene (Fig. 1*A*). These data suggest that comparing mammalian, reptile, and bird alleles would provide a broad consistent background for determining which paralog-specific amino acid differences are conserved.

We next looked for candidate amino acids that might lead to divergent functions. Primary sequence alignment of human SAR1A and SAR1B found three clusters where three or more amino acids diverged in close proximity. Of those three clusters, two were highly conserved among mammals, reptiles, and birds (106–117 and 139–146) (Fig. 1*B*), whereas one (162–164) was less conserved.

SAR1 residues 139–146 are adjacent to the GTP-binding pocket, and residues 106–117 are on an α-helix near the known binding site of SEC31 on SEC23 (Fig. 1, *C* and *D*). Notably, the divergent amino acids on the α-helix all appear on the exposed surface of the protein, suggesting that they may have a role in protein–protein interactions. The importance of the interaction between SEC31 and the SAR1 GTPase cycle for large cargo secretion has been well documented (6, 20–22). We hypothesized that the GTP-adjacent cluster of divergent residues may play a role in GTP exchange or hydrolysis, whereas the SEC23/31-adjacent divergent α-helix may play a role in binding SEC31.

### SAR1A has faster GTPase exchange than SAR1B

To test whether the divergent GTP-adjacent amino acids led to different GTP cycle activity in SAR1, we utilized a tryptophan fluorescence–based assay performed with purified human proteins (23–25). In this assay, the nucleotide-bound state of Sar1 is monitored by relative fluorescence measurements. The intrinsic tryptophan fluorescence of SAR1-GTP is significantly higher than that of SAR1-GDP.

**Figure 2.**
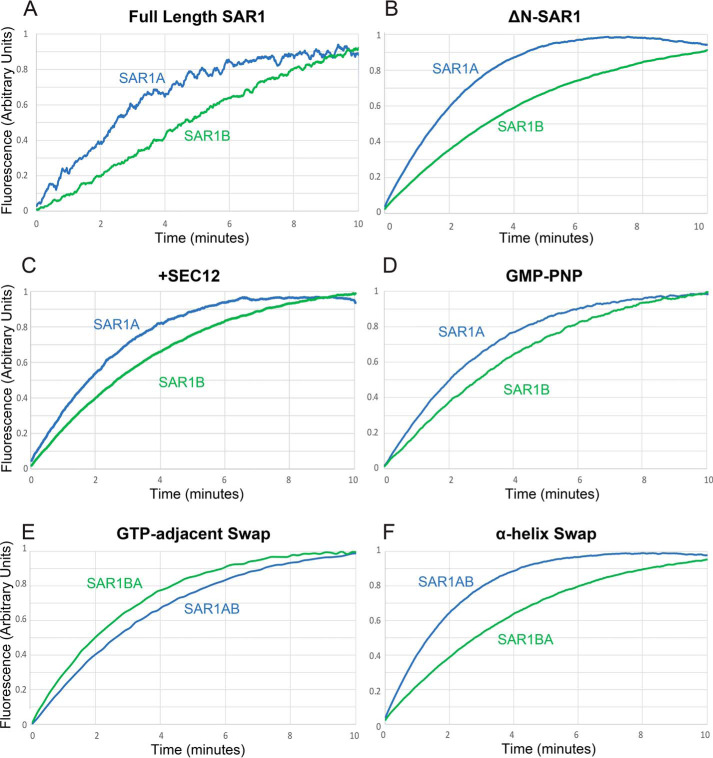
**GTP exchange in SAR1A and SAR1B.** *A–F*, tryptophan fluorescence assay measuring loading of GTP into SAR1A and SAR1B with full-length SAR1 and liposomes (*A*), ΔN-SAR1 (*B*), ΔN-SAR1 and SEC12 (*C*), ΔN-SAR1 and nonhydrolyzable GMP-PNP in place of GTP (*D*), ΔN-SAR1 with the GTP-adjacent divergent amino acid cluster swapped (*E*), and ΔN-SAR1 with the SEC31-binding site adjacent divergent amino acid cluster swapped (*F*).

We first analyzed the kinetics of GTP exchange upon addition of GTP to the reaction. We found that, with full-length SAR1 in the presence of liposomes, SAR1A loaded significantly faster than SAR1B (Fig. 2*A*). This difference applied to soluble forms of SAR1 that lack the N-terminal amphipathic helix and, as a result, do not require liposomes (Fig. 2*B*). Addition of the SAR1 guanine exchange factor SEC12 proportionally increased the loading speed of both paralogs (Fig. 2*C*). We confirmed that this effect was unrelated to hydrolysis by substitution of a nonhydrolyzable GTP analog 5′-Guanylyl imidodiphosphate (GMP-PNP) (Fig. 2*D*).

We hypothesized that this difference may be due to the GTP-adjacent divergent residues. To test this, we purified SAR1 proteins with the divergent amino acids swapped between paralogs (SAR1A>B-GTPa and SAR1B>A-GTPa). We found that changing these three amino acids reversed the kinetic differences between SAR1A and SAR1B (Fig. 2*E*). Conversely, we found that constructs in which the amino acids of the divergent apical α-helix (SAR1A>B-helix and SAR1B>A-helix) were swapped did not reverse the kinetics (Fig. 2*F*). These data suggest that the GTP-adjacent divergent residues are necessary and sufficient for the increased kinetics of SAR1 GTP exchange.

Having observed differences in GTP loading, we probed the SAR1 paralogs for GTP hydrolysis using the tryptophan fluorescence assay. We did not find a strong consistent difference between the two paralogs in the presence of different sizes of liposomes (Fig. S1*A*) or SEC23A or SEC23B (Fig. S1*B*). This evidence suggests that, although SAR1A exchanges nucleotide more quickly that SAR1B, GTP hydrolysis may be unaffected, at least *in vitro*.

### SAR1A binds the GTPase-activating fragment of SEC31 more strongly that SAR1B

**Figure 3.**
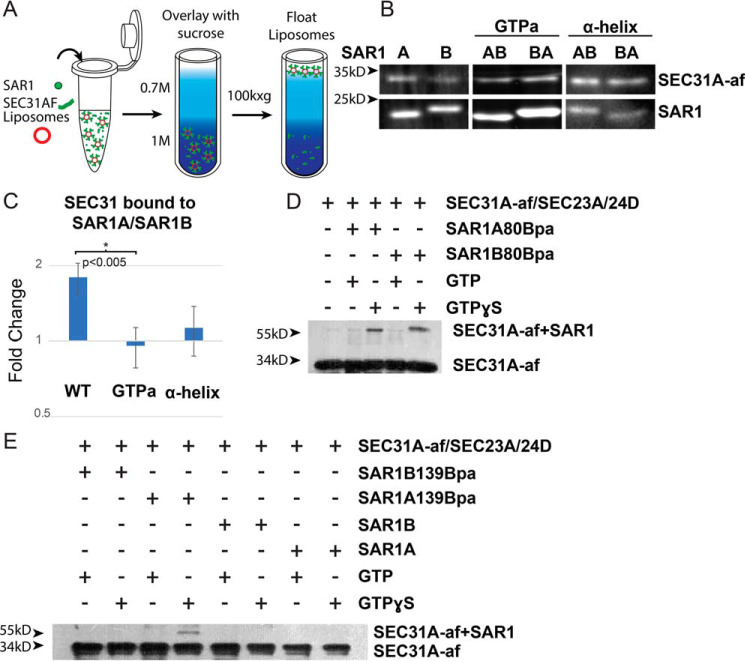
**Binding of SAR1 paralogs and the SEC31A GTPase-activating fragment.** *A*, schematic of the liposome flotation assay. COPII proteins are incubated with liposomes and then floated through a sucrose gradient to determine which proteins bind to liposomes. *B*, SDS-PAGE followed by SYPRO-Ruby staining of SEC31A-af recruited by SAR1 to floated liposomes. *C*, quantification of the liposome flotation experiments with the SEC31-af signal normalized to SAR1 (*n* > 3 for each condition). *D* and *E*, photo-cross-linking assay of SAR1 with an unnatural amino acid at positions 80 (*D*) and 139 (*E*) incubated with SEC31A-af and photoactivated. The *upper band* indicates cross-linked SAR1/SEC31A-af.

One of the two divergent clusters between SAR1A and SAR1B is adjacent to the known SEC31/SEC23 binding site. We therefore hypothesized that this cluster may be important for direct binding of SEC31 to SAR1. To test the extent of direct binding between SAR1 and SEC31, we utilized a liposome flotation assay. Purified recombinant human SAR1 and the GTPase-activating fragment of SEC31A (SEC31A-af) were incubated with synthetic liposomes and GMP-PNP. The reaction was applied to the bottom of a sucrose density gradient. After a high-speed centrifugation step, liposomes carried bound SAR1 and any SEC31 bound to that SAR1 with them as they floated to the top of the sucrose gradient (Fig. 3*A*). We then evaluated the levels of SAR1 and SEC31A-af present on the liposomes by SDS-PAGE followed by SYPRO-Ruby staining.

We found that SAR1A recruited SEC31A-af ∼2-fold more efficiently than SAR1B (Fig. 3, *B* and *C*). When we performed the same assay with SAR1A>B-helix and SAR1B>A-helix, however, the difference became negligible, suggesting that the divergent α-helix played a role in SEC31 binding but was not the sole reason for differences between SAR1A and SAR1B.

Here we made a secondary observation. Despite having similar molecular weights, SAR1B migrated more slowly in SDS-PAGE gels than SAR1. However, SAR1A>B-helix migrated similarly as SAR1B and SAR1B>A-helix similarly as SAR1A (Fig. 3*B*). Therefore, whatever causes this different migration of the two paralogs is contained in the α-helix.

We then compared SAR1A>B-GTPa and SAR1BA-GTPa. Unexpectedly, swapping the GTP-adjacent divergent residues had a more significant effect than swapping the α-helix (Fig. 3, *B* and *C*), suggesting that the GTP-adjacent residues have as much of a role, if not more, than the divergent α-helix in SEC31 binding.

Because the portion of SEC31A that has been resolved by crystallography is relatively small, the extent of the binding between SEC31 and SAR1 is unknown. To confirm whether the GTP-adjacent residues directly bind SEC31A-af, we used photo-cross-linking to an unnatural amino acid in recombinant SAR1 protein. We verified that the assay detected SEC31A-af binding by inserting an unnatural amino acid at position 80, directly adjacent to the known SEC23A/SEC31A-af binding site. Cross-linking was stimulated by incubation with the nonhydrolyzable GTP analog GTPγS and either SAR1 paralog (Fig. 3*D*). We then repeated this by inserting the unnatural amino acid at residue 139 (threonine in SAR1A and proline in SAR1B). We chose this residue because it is the part of the GTP-adjacent cluster that is most distant from the known SEC23A/SEC31A binding site, and, therefore, background binding is likely to be low. Cross-linking was stimulated by GTPγS primarily in SAR1A (Fig. 3*E*), consistent with our liposome flotation data. These data suggest that both divergent clusters play a role in SAR1–SEC31 binding and that SAR1A has a higher affinity for direct binding of SEC31.

### SAR1B binds SEC23 more strongly than SAR1A

Although our data show that SAR1 can directly bind SEC31, SAR1 normally binds SEC31 in conjunction with SEC23. Using the liposome flotation assay, we compared recruitment of SEC23 and SEC31 by the two SAR1 paralogs. Because SEC23A and SEC23B also have paralog-specific roles in large cargo secretion, we also tested whether SAR1 had a different affinity for SEC23A or SEC23B by adding both in competition to the reaction.

**Figure 4.**
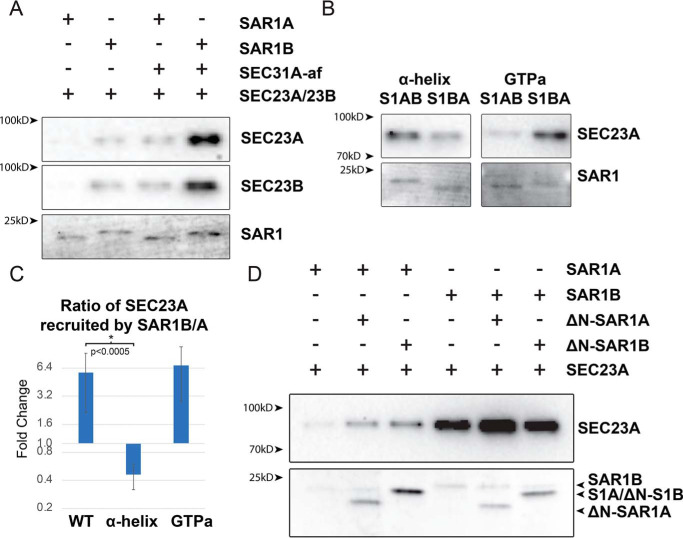
**Binding of SAR1 and SEC23.** *A* and *B*, SDS-PAGE followed by transfer to a PVDF membrane and SYPRO-Ruby staining of SAR1 WT (*A*) or the indicated divergent amino acid group swaps (*B*) and immunoblot of the indicated SEC23 paralog recruited to liposomes by SAR1. *C*, quantification of the liposome flotation experiments with the SEC23A signal normalized to SAR1 (*n* > 3 for each condition). *D*, SDS-PAGE followed by SYPRO-Ruby staining of the indicated SAR1 and ΔN-SAR1 recruited to liposomes by SAR1 and immunoblot of SEC23A recruited to liposomes by SAR1.

We found a strong divergence between SAR1A and SAR1B in their recruitment of either paralog of SEC23. SAR1B recruits SEC23 at an ∼5-fold higher level than SAR1A (Fig. 4, *A* and *C*). Addition of SEC31A-af increased the binding of both SAR1 paralogs for both SEC23 paralogs, but the binding of SAR1B to SEC23 remained ∼5-fold higher than SAR1A.

To determine which amino acids in SAR1 were responsible for the divergence in SEC23 recruitment, we repeated the flotation assay with the GTP-adjacent and helix SAR1 variants. We found that swapping the divergent α-helix reversed affinity for SEC23, reducing SAR1B>A-helix binding to SEC23 by ∼2-fold instead of a 5-fold increase (Fig. 4, *B* and *C*). These data suggest that the divergent α-helix leads to SAR1B having a higher affinity for SEC23.

We hypothesized that SAR1B's greater affinity for SEC23 may interfere with SAR1A's ability to recruit SEC23 and the remaining elements of the COPII coat if it were present in excess over SAR1A. To test this, we performed the liposome flotation assay using the soluble N-terminally deleted SAR1. We hypothesized that addition of soluble SAR1B may decrease the amount of SEC23 recruited by SAR1A by sequestering the available pool of protein. However, we found the opposite: addition of either soluble SAR1 paralog enhanced SEC23 recruitment (Fig. 4*D*).

Enhanced recruitment of SEC23 may be due to formation of SAR1 dimers on the membrane (26–28). In fact, we found a significant amount of soluble SAR1 recruited to the membrane, although we were unable to distinguish soluble SAR1B from SAR1A, as the shortened SAR1B and the full-length SAR1A have the same SDS-PAGE mobility. SAR1A appeared to be prone to recruiting soluble SAR1 more than SAR1B (Fig. 4*D*). These data suggest that SAR1A may have a higher affinity for homodimerization than SAR1B.

### SAR1A homodimerizes more strongly than SAR1B

To evaluate oligomerization of SAR1 on a membrane surface, we developed a liposome aggregation assay. We hypothesized that SAR1 dimerization could cause small liposomes to aggregate, decreasing the number of particles and increasing their size.

**Figure 5.**
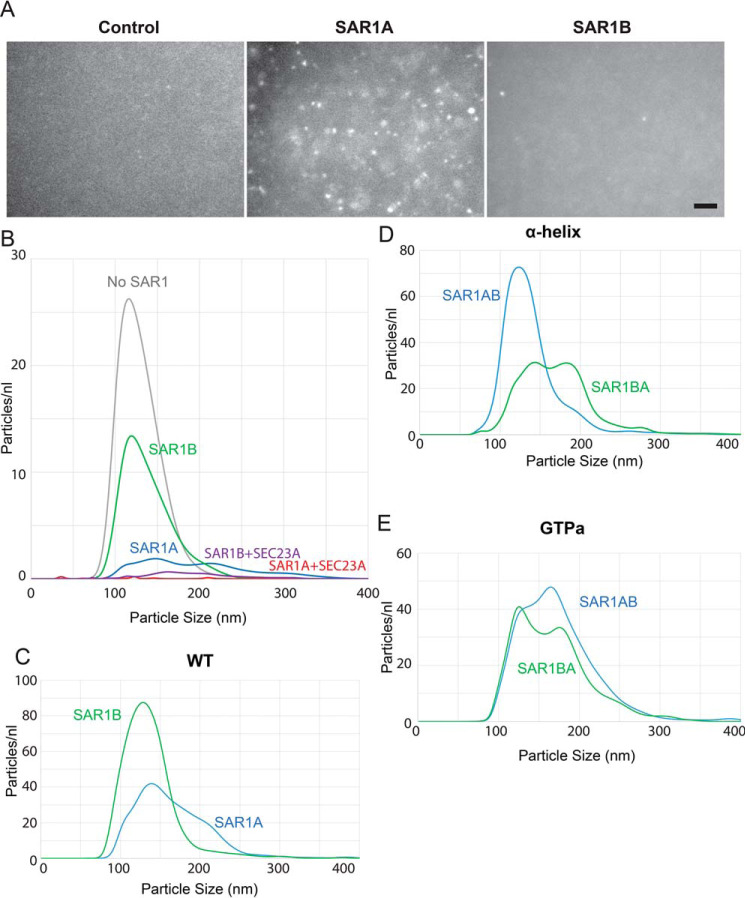
**Liposome aggregation by SAR1 paralogs.** *A*, fluorescence microscopy of 100-nm liposomes incubated overnight at 37 °C. Only aggregates of more than 200 nm can be clearly resolved. *Scale bar* = 10 μm. *B–E*, nanoparticle analysis of 100-nm liposomes incubated with GMP-PNP and the indicated proteins. Particle count is inversely correlated to aggregation.

We prepared small 100-nm synthetic liposomes and incubated them with SAR1 and GTP-PNP. After overnight incubation, SAR1A-containing liposomes formed aggregates easily visible by light microscopy (Fig. 5*A*), whereas SAR1B-containing liposomes were indistinguishable from liposomes alone. The size and number of particles in the suspension were then evaluated with a Nanosight particle analyzer. To detect smaller particles that could be reliably quantified by Nanosight, we used lower protein concentrations and shorter incubation times. Under these conditions, nanoparticle tracking allowed us to see a difference between liposomes alone and liposomes with SAR1B. SAR1B-containing liposomes were larger on average and fewer in number than liposomes alone, suggesting that SAR1B does undergo some homodimerization. The change in particle number and size is more substantial with SAR1A (Fig. 5*B*). Addition of SEC23/24 heterodimer greatly increased the size of particles and reduced their number (Fig. 5*B*), as expected, because SEC23 binds directly to SAR1A and SAR1B, and the heterodimers bind each other.

To test whether this effect was due to either of the divergent peptide clusters, we repeated this assay with the WT (Fig. 5*C*), helix (Fig. 5*D*), and GTP-adjacent cluster (Fig. 5*E*) SAR1 constructs. We found that swapping the divergent α-helix caused SAR1BA-helix to promote liposome aggregation more than SAR1AB-helix (Fig. 5*D*), suggesting that the divergent α-helix is the primary driver of SAR1A oligomerization/aggregation. We found that swapping the GPTa cluster also resulted in increased aggregation by SAR1BA-GTPa (Fig. 5*E*). This effect is milder than that seen with SAR1BA-helix, suggesting that the GTP-adjacent cluster may also play a role in oligomerization but a relatively minor one compared with the apical α-helix. These data suggest that SAR1A oligomerizes on a membrane surface and the divergent α-helix has a more prominent role in this association than the GTP-adjacent cluster.

### The divergent α-helix in SAR1B facilitates rescue of lipoprotein secretion

**Figure 6.**
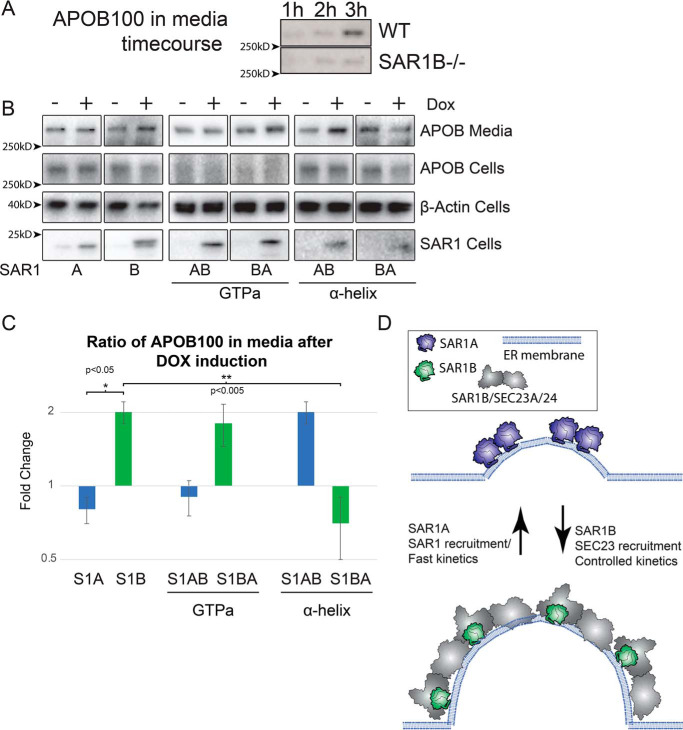
**Apolipoprotein B 100 secretion and SAR1.** *A*, immunoblot of APOB in the top fraction of OptiPrep gradient from medium of WT and SAR1B^−/−^ McArdle cells incubated with oleic acid for the indicated time. *B*, immunoblot of APOB in SAR1B^−/−^ McArdle cells transformed by a lentivirus to overexpress the indicated SAR1 constructs after doxycycline induction. β-Actin was used as a loading control. *C*, quantification of APOB secreted into medium (*n* = 3). *D*, proposed model of divergent roles of SAR1 paralogs in cells. SAR1A is proposed to homodimerize at the membrane for ER exit site remodeling, whereas SAR1B, which has a higher affinity for SEC23, leads to higher recruitment of the SEC23/24 heterodimer, enabling better secretion of large cargos that rely on tighter control of the kinetics of COPII vesicle formation.

To test the functionally significant differences of the SAR1 paralogs in cells, we measured apolipoprotein secretion in transfected cells. For this purpose, we developed CRISPR-Cas9–mediated SAR1B knockdown with the lipoprotein-secreting rat hepatoma cell line McArdle RH7777. Cells were incubated in oleic acid–containing medium from which samples were withdrawn every 1–3 h. Aliquots of medium were subjected to density sedimentation on an OptiPrep gradient to collect the buoyant lipoproteins. Secreted APO1B was detected and quantified by immunoblot. As has been found previously (29), loss of SAR1B resulted in a substantial reduction of APOB100 in the medium (Fig. 6*A*).

Using lipoprotein secretion–deficient SAR1B^−/−^ cells, we generated inducible cell lines using a lentivirus and a tetracycline repressor system to control the expression of SAR1 by addition of doxycycline. We found that overexpression of SAR1B increased the amount of APOB100 secreted into the medium whereas overexpression of SAR1A did not (Fig. 6*B*). Overexpression of the GTP-adjacent swap SAR1B>A produced an ∼2-fold increase in ApoB secretion, similar to the effect of WT SAR1B, suggesting that the GTP-adjacent amino acid cluster is not relevant to the SAR1B-specific function. Overexpression of the α-helix swap SAR1B>A failed to increase APOB100 secretion, whereas SAR1A>B increased ApoB secretion ∼2-fold, suggesting that the α-helix is most important for the paralog-specific function of SAR1B in lipoprotein secretion.

Taken together, these data suggest that SAR1A and SAR1B differ biochemically. The paralogs have two divergent clusters of amino acids, one adjacent to the GTP binding pocket and one in an α-helix on the apical side of the protein, that have conserved paralog–specific sequences and functions. The GTP-adjacent cluster causes SAR1A to exchange GTP more rapidly than SAR1B. The apical α-helix cluster causes SAR1B to have a higher affinity for SEC23A and has an important role in lipoprotein secretion.

## Discussion

Vertebrate cells have a wide variety of distinct secretory requirements in the types of cargo and the overall cargo load. Evolution of different paralogs of the basic machinery gives cells specialized tools to deal with the particular needs of a given cell at a given time. We found how small changes in the primary sequence of SAR1 paralogs lead to different biochemical characteristics that have significant effects on cellular function.

### SAR1 as a remodeler and cargo size

It is easy to think of SAR1 as just a small part of a bigger COPII machinery rather than focusing on the important roles of SAR1 itself. Hanna *et al.* (27) showed that the amphipathic helix of GTP-bound Sar1 stably penetrates the endoplasmic reticulum membrane, promoting local membrane deformation. As membrane bending increases, Sar1 membrane binding is elevated, ultimately culminating in GTP hydrolysis, which may destabilize the bilayer sufficiently to facilitate membrane fission. Lee *et al.* (30) showed that SAR1 promotes significant membrane remodeling by itself, a finding that has been amplified by compelling atomic force microscopy videos of rapid rearrangement of the membrane induced by SAR1. SAR1 dimerization may aid in membrane remodeling (26–28).

Recent cryotomography (31) of COPII assembled on the membrane suggests quite the opposite situation. Hutchings *et al.* (31) present data showing an elegant, ordered array of SAR1/SEC23/SEC24 subunits on a tubular membrane with no evidence of SAR1 dimerization in the assembled COPII structures. The authors propose that COPII forms small vesicles using inner coat patches that insert Sar1 amphipathic helices randomly and curve the membrane in all directions, similar to what was seen with atomic force microscopy. They propose that larger structures, in contrast, are formed by extensive assembly of the inner coat, consistent Sar1 orientation, and parallel insertion of its amphipathic helix. Taking these results together, and having identified biochemical differences between SAR1 paralogs, we propose the following model for how the SAR1 paralogs might play a role in cells (Fig. 6*D*).

### A bimodal model of SAR1 and COPII behavior

Trafficking of large cargos presents a special challenge to the COPII machinery. Disruption of transcriptional regulators of COPII can also lead to large cargo–specific defects (32–34), suggesting that large cargos are especially sensitive to changes in COPII dynamics. The kinetics of COPII assembly have been found to be especially important for trafficking of collagen and large lipoproteins (21, 22, 35). In collagen trafficking, for example, TANGO1 competes with SEC31 for binding to SEC23, postponing the final steps of budding until collagen is loaded into vesicles (22, 35). Conversely, for small cargos, faster kinetics would allow cells to more quickly address their trafficking needs.

We propose that, under conditions where speed is more critical than size, such as when small cargos need to be trafficked, SAR1 dimers insert their amphipathic helix into the membrane, creating high levels of curvature, and thus recruit more SAR1 to the membrane through dimerization and an affinity for a highly curved membrane. This leads to numerous small vesicles and fast budding. This process may be most efficiently driven by SAR1A, with its fast GTP loading and efficient oligomerization (Fig. 6*C*, *top*).

Under conditions where speed is less critical than size, such as large cargo secretion, SAR1 recruits SEC23/24 heterodimers and forms ordered lattices that allow slower, more controlled membrane curvature and packaging of large cargos. This process may be most efficiently driven by SAR1B, with its high affinity for SEC23 and less efficient oligomerization (Fig. 6*C*, *bottom*). In this model, controlling the balance of the two SAR1 paralogs would give cells a mechanism to respond to different secretory requirements.

### Paralog-specific functions not unique to SAR1

The existence of multiple paralogs of mammalian COPII subunits provides an opportunity for fine-tuning of cargo sorting dependent on the physiologic needs of different cells and tissues. In addition, COPII protein levels are dynamically regulated (32, 33). Loss of function of these paralogs leads to many distinct phenotypes (36–43). In an extreme example, SEC13 has a dual role as a nuclear pore component and as a subunit of the outer shell of the COPII coat (44).

The unique roles of COPII paralogs present a dynamic picture of COPII regulation of and response to protein trafficking and other cellular needs. Rather than a single complex with a single purpose, COPII paralogs provide a cellular membrane trafficking toolkit.

## Experimental procedures

### Phylogenetic analysis

Protein sequences were aligned using ClustalOmega (RRID:SCR_001591). A cladogram tree was constructed using Clustal alignment.

### Antibodies

Commercially available antibodies used for immunoblotting were as follows: goat anti-apolipoprotein B (EMD, Hayward, CA, USA; 178467, 1:500, for immunoblot; Fig. 6*A*) and rabbit anti-apolipoprotein B (Proteintech, Rosemont, IL, USA; 20578-1-AP; Fig. 6*B*).

### Lentivirus production and adipocyte transduction

Human SAR1 was subcloned into pLenti-puro (Addgene, Cambridge, MA, USA; plasmid 39481). The plasmid containing SAR1 was transfected with Lipofectamine 2000 (Invitrogen) following the manufacturer's recommendations into HEK293T cells at 50% confluence on the day of transfection along with the lentiviral packaging plasmids pVSVg (3.5 μg) and psPAX2 (6.5 μg, Addgene). Transfection was performed using one 10-cm dish. After 24-h transfection, the medium was changed, and after an additional 24 h, the medium was removed and filtered through a 0.45-μm low-protein binding membrane (VWR International, Radnor, PA, USA). McArdle-RH7777 or IMR-90 cells were then transduced with the virus with 8 μg/ml Polybrene (Sigma-Aldrich). After 24 h, the medium was replaced with fresh medium, and after an additional 24 h, 2 μg/ml puromycin (Sigma-Aldrich) was added to select transduced cells.

### Protein purification

Human SAR1- and SEC31-GTP–activating fragment proteins were expressed in *Escherichia coli*, purified as GST fusions, and then cleaved, as described for hamster Sar1 purification (45). In brief, a bacterial lysate was first centrifuged at 43,000 × *g* for 15 min, and then the supernatant fraction was further centrifuged at 185,000 × *g* for 1 h. The supernatant was incubated with prewashed GSH-agarose (1 ml slurry/liter bacteria; Thermo Fisher Scientific) for 1 h at 4 °C. Agarose was washed with wash buffer (50 mm Tris (pH 7.4), 150 mm NaCl, 0.1% Tween, 5 mm MgCl_2_, and 100 μm GDP), and protein was eluted by cleaving with 20 units/ml thrombin (Roche) in TCB (50 mm Tris (pH 8), 250 mm KoAc, 5 mm CaCl_2_, 5 mm MgCl_2_, and 100 μm GDP). Human SEC23/24 paralogs and variants were purified from lysates of baculovirus-infected insect cells as described previously (45). In brief, insect cell lysates were centrifuged at 185,000 × *g* for 1 h, and 30% ammonium sulfate was added to the supernatant fraction at 4 °C. The precipitant was collected by centrifugation at 30,000 × *g* for 30 min and solubilized in no-salt buffer (20 mm Hepes (pH 8), 10% glycerol, 250 mm sorbitol, 0.1 mm EGTA, 5 mm β-mercaptoethanol, and 10 mm imidazole). The solubilized 30% ammonium sulfate precipitant was cleared at 30,000 × *g* for 20 min, and the supernatant was incubated with prewashed Ni-NTA resin (1.25 ml slurry/liter insect cells, Thermo Fisher Scientific) for 1 h at 4 °C. Ni-NTA was washed with 20 mm Hepes (pH 8), 10% glycerol, 250 mm sorbitol, 500 mm KoAc, 0.1 mm EGTA, 5 mm β-mercaptoethanol, and 50 mm imidazole and eluted with 250 mm imidazole. Ni-NTA–eluted SEC13/31A protein was further purified using an anion exchange column (MonoQ) on an AKTA FPLC system (GE Healthcare).

### Immunoblotting

Standard immunoblotting procedures were followed. In brief, samples were resolved on 4%–20% polyacrylamide gels (15-well, Invitrogen; 26-well, Bio-Rad) and transferred to PVDF (EMD Millipore) at constant 0.5 A for 4 h. The PVDF membrane was incubated with antibodies (primary overnight at 4 °C h and secondary for 1 h at room temperature), and bound antibodies were visualized by the enhanced chemiluminescence method (Thermo Fisher Scientific) on a ChemiDoc imaging system (Bio-Rad) with ImageLab software v4.0 (Bio-Rad).

### Liposome binding assay

The liposome binding assay was performed as described for yeast COPII proteins (45, 46) using 10% cholesterol major–minor mixture liposomes with Texas Red^TM^ 1,2-dihexadecanoyl-sn-glycero-3-phosphoethanolamine, triethylammonium salt (Thermo Fisher Scientific) for visualization. Liposomes were extruded through a polycarbonate filter with 100-nm pore size (Whatman). Following a 30-min incubation at 37 °C, the protein–liposome mixture was diluted into 2.5 m sucrose in HKM (20 mm Hepes-K, pH 6.8, 160 mm KOAc, 1 mm MgCl_2_) buffer to a final concentration of 1 m sucrose. The sample was overlaid with 100 μl of 0.7 m sucrose and then 20 μl of HKM and separated by centrifugation at 391,000 × *g* for 4 h at 4 °C.

### Liposome aggregation assay

For visualization by microscopy, each 50-μl reaction contained 2 μg of SAR1 and ∼30,000 particles/μl liposomes in HKM buffer. Samples were incubated overnight at 37 °C, directly pipetted onto a coverslip, and imaged with Zeiss Axiovision using the Texas Red fluorescent filter. For the nanoparticle tracking analysis, the 50-μl reaction contained 1 μg of SAR1 and ∼30,000 particles/μl liposomes in HKM buffer incubated for 3 h at 37 °C. Samples were diluted 1:1000 before analysis.

### GTPase activity assay

The tryptophan fluorescence GTPase activity assay was performed at 37 °C as described previously (23–25) using a stirred-cell cuvette. In HKM buffer, we added soluble SAR1B to a final concentration of 1.33 μm and, where indicated, SEC31 active fragment (25) (2 μm). Five minutes later, GTP was added to 30 μm. After exchange of GDP for GTP was complete (∼10 min), SEC23–SEC24D complex was added to 250 nm.

### Nanoparticle tracking analysis

Sizes of vesicles budded *in vitro* were estimated using the NanoSight NS300 instrument equipped with a 405-nm laser (Malvern Instruments, Malvern, UK). Particles were analyzed in scatter mode without a filter. 100-nm silica microspheres (Polysciences, Warrington, PA, USA) were analyzed to check instrument performance and determine the viscosity coefficient of B88. Aliquots (1 μl) of vesicles diluted 1000× with 999 μl of filtered B88 (0.02 μm, Whatman). The samples were automatically introduced into the sample chamber at a constant flow rate of 50 (arbitrary manufacturer unit, ∼10 μl/min) during five repeats of 60-s captures at camera level 11 in scatter mode with Nanosight NTA 3.1 software (Malvern Instruments). The particle size was estimated with detection threshold 5 using Nanosight NTA 3.1 software, and then “experiment summary” and “particle data” were exported. Particle numbers in each size category were calculated from the particle data, in which “true” particles with a track length of more than 3 were pooled, binned, and counted with Excel (Microsoft).

### Generation of CRISPR-Cas9 KO cell lines

McArdle-RH7777 cells were transfected with a pX330 vector–derived plasmid (47) containing the targeting sequence from SAR1B (GATGTAGTGTTGGGACGTGCTGG) and a PGK1 promotor–driven Venus construct (reconstructed by Liangqi Xie from Robert Tjian's laboratory at University of California, Berkeley). After 24-h transfection, FACS was performed to inoculate single transfected cells in each well of 96-well plates. After 2 weeks, single colonies were expanded and validated by immunoblot and DNA sequencing of the targeted area. Validated positive colonies were employed for the experiments.

### Statistical analysis

Data in bars represent average ± S.D. Statistical analyses on qualitative data were performed using analysis of variance followed by post hoc Holm *p* value adjustment. Statistical analyses on categorical data were performed using chi-square test followed by post hoc pairwise Fisher's exact test with Holm *p* value adjustment using the R statistical package.

### Cloning and expression of SAR1 for the cross-linking assay

Unnatural amino acids were incorporated into Sar1A or Sar1B at the noted positions using the following method. pEVOL-pBpf, coding for the tRNA synthetase/tRNA pair, was used for *in vivo* incorporation of *p-*benzoyl-l-phenylalanine into proteins at the position of an in-frame amber stop codon (TAG) (48, 49). The mutant Sar1 paralogs containing TAG codons were coded for the pGEX-2T vector. The noted plasmids were cotransformed into DH10B-competent cells in a pulser cuvette. The cells were plated and grown overnight at 37 °C on lysogeny broth agar containing chloramphenicol and ampicillin. Colonies were picked and grown at 37 °C in lysogeny broth containing ampicillin/chloramphenicol to an *A*_600_ of ∼0.4. The temperature was changed to 25 °C, and protein expression was induced with 1 mm isopropyl 1-thio-β-d-galactopyranoside and 0.02% arabinose in the presence of 1 mm *p-*benzoyl-l-phenylalanine. Cells were harvested after 16 h, and the protein was purified as described previously (16). The proteins were analyzed by electrospray ionization MS to verify unnatural amino acid incorporation.

### Photoactivated cross-linking

Photo-cross-linking was performed by incubating Sar1A or B (1 μg), 1 mm GTP or GTPγS, 2.5 μg of Sec23A/Sec24D, 2.5 μg of Sec13/31A, and 1.7 μg of liposomes at 32 °C for 30 min. Samples were placed in a 96-well flat-bottom plate and irradiated using a handheld UV lamp (∼360 nm) at 4 °C for 5 min. The samples were removed from the wells, resolved on an SDS-PAGE gel, transferred to nitrocellulose, and detected by immunoblotting with an antibody recognizing Sec31A.

## Data availability

All data are contained in this manuscript.

## Supplementary Material

Supporting Information
