# Supplementary material for: Small sequence variations between two mammalian paralogs of the small GTPase SAR1 underlie functional differences in coat protein complex II assembly
Source: J Biol Chem. 2020 May 1;295(25):8401–12. doi: 10.1074/jbc.RA120.012964 (PMC7307210; doi:10.1074/jbc.RA120.012964)
Supplement: Supporting Information [file supp_295_25_8401__index.html]

Small sequence variations between two mammalian paralogs of the small GTPase SAR1 underlie functional differences in coat protein complex II assembly — SAR1 paralogs differ biochemically — Supporting Information 

# Small sequence variations between two mammalian paralogs of the small GTPase SAR1 underlie functional differences in coat protein complex II assembly

## Supporting Information

- Supplemental Figure 1 - Supplemental Figure 1
