# Supplementary figures and images for: Small sequence variations between two mammalian paralogs of the small GTPase SAR1 underlie functional differences in coat protein complex II assembly
Source: J Biol Chem. 2020 May 1;295(25):8401–12. doi: 10.1074/jbc.RA120.012964 (PMC7307210; doi:10.1074/jbc.RA120.012964)

A

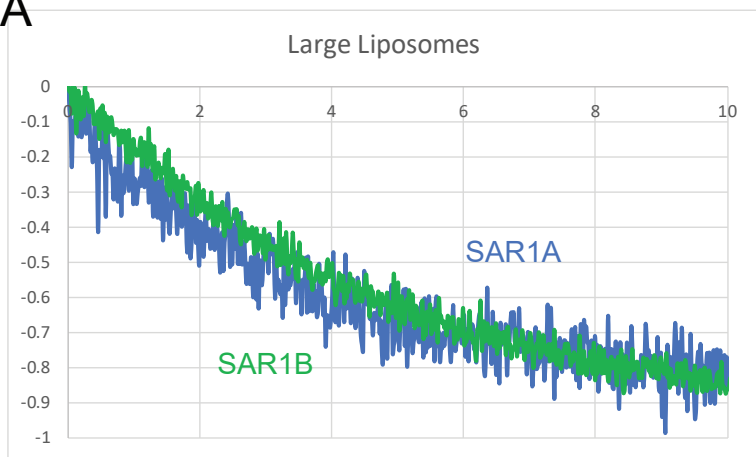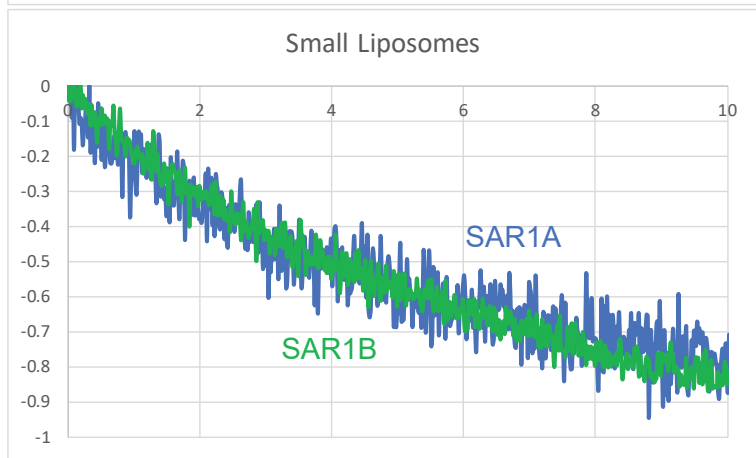

B

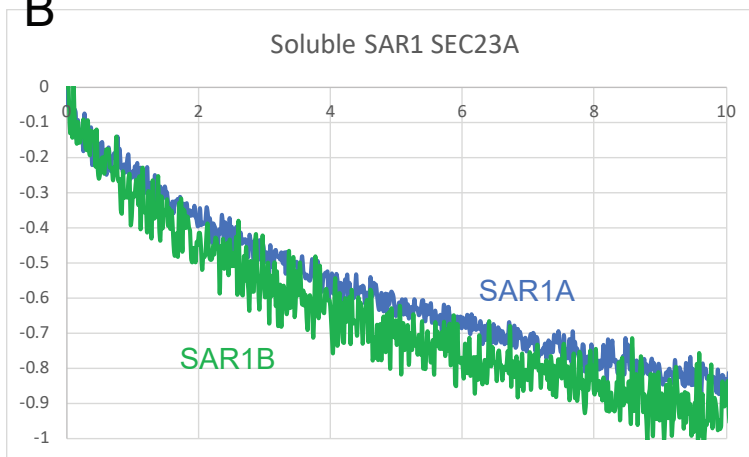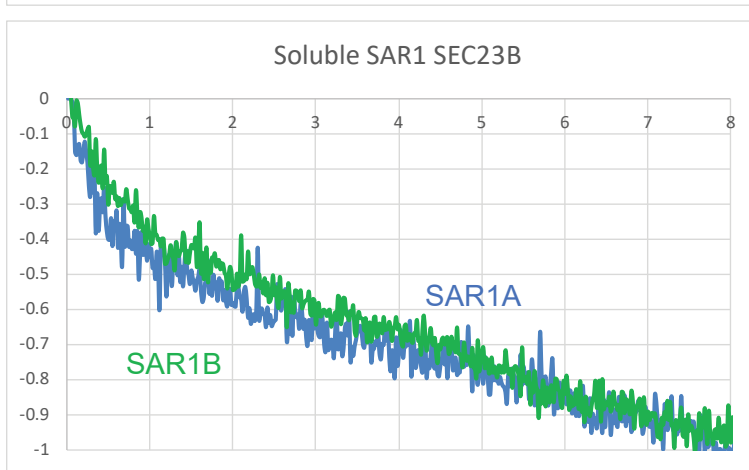

Supplement: Supporting Information [file supp_RA120.012964_158711_2_supp_519924_q9h021.pdf]
